# Impact of COVID-19 Pandemic on Social Determinants of Health Issues of Marginalized Black and Asian Communities: A Social Media Analysis Empowered by Natural Language Processing

**DOI:** 10.1007/s40615-024-01996-0

**Published:** 2024-04-16

**Authors:** Christopher Whitfield, Yang Liu, Mohd Anwar

**Affiliations:** North Carolina A&T State University, Greensboro, NC 27411 USA

**Keywords:** COVID-19, Social determinants of health (SDOH), Health disparities, Black communities, Social media, Natural language processing (NLP), Machine learning (ML)

## Abstract

**Purpose:**

This study aims to understand the impact of the COVID-19 pandemic on social determinants of health (SDOH) of marginalized racial/ethnic US population groups, specifically African Americans and Asians, by leveraging natural language processing (NLP) and machine learning (ML) techniques on race-related spatiotemporal social media text data. Specifically, this study establishes the extent to which Latent Dirichlet Allocation (LDA) and Gibbs Sampling Dirichlet Multinomial Mixture (GSDMM)-based topic modeling determines social determinants of health (SDOH) categories, and how adequately custom named-entity recognition (NER) detects key SDOH factors from a race/ethnicity-related Reddit data corpus.

**Methods:**

In this study, we collected race/ethnicity-specific data from 5 location subreddits including New York City, NY; Los Angeles, CA; Chicago, IL; Philadelphia, PA; and Houston, TX from March to December 2019 (before COVID-19 pandemic) and from March to December 2020 (during COVID-19 pandemic). Next, we applied methods from natural language processing and machine learning to analyze SDOH issues from extracted Reddit comments and conversation threads using feature engineering, topic modeling, and custom named-entity recognition (NER).

**Results:**

Topic modeling identified 35 SDOH-related topics. The SDOH-based custom NER analyses revealed that the COVID-19 pandemic significantly impacted SDOH issues of marginalized Black and Asian communities. On average, the Social and Community Context (SCC) category of SDOH had the highest percent increase (366%) from the pre-pandemic period to the pandemic period across all locations and population groups. Some of the detected SCC issues were racism, protests, arrests, immigration, police brutality, hate crime, white supremacy, and discrimination.

**Conclusion:**

Reddit social media platform can be an alternative source to assess the SDOH issues of marginalized Black and Asian communities during the COVID-19 pandemic. By employing NLP/ML techniques such as LDA/GSDMM-based topic modeling and custom NER on a race/ethnicity-specific Reddit corpus, we uncovered various SDOH issues affecting marginalized Black and Asian communities that were significantly worsened during the COVID-19 pandemic. As a result of conducting this research, we recommend that researchers, healthcare providers, and governments utilize social media and collaboratively formulate responses and policies that will address SDOH issues during public health crises.

## Introduction

The COVID-19 pandemic has contributed to a sharp increase in health disparities among marginalized US populations. This is highly significant as nearly 40% of the people in the United States population today identify as racial or ethnic minorities.^1^ Roughly 19.1% are Latino, 13.6% are African American, and 6.3% are Asian.^2^ Hence, about four out of ten Americans are racial/ethnic minorities who are at-risk at being disproportionately affected by a public health crisis. As the newest public health crisis, the COVID-19 pandemic has brought many public health inequities to the forefront, highlighting ways that COVID-19 has unequally affected many racial and ethnic minority groups. For example, among US deaths per 100,000 people as of March 7, 2021, 178 African Americans, 172 Native Americans, and 154 Latinos have died due to COVID-19. With Whites accounting for 124 deaths, African Americans died at a rate of 1.4 times their White counterparts.^3^ This mortality instance is just one of many statistics showing how marginalized racial and ethnic groups might be disproportionately affected by the COVID-19 pandemic.

Moreover, given the disproportionate impact of the COVID-19 pandemic on marginalized communities, it is imperative to explore avenues for understanding and addressing these disparities. Consequently, the utilization of social media platforms for public health surveillance research has garnered increasing attention [1–3]. Since the advent of the COVID-19 pandemic, people have turned to social media to express opinions, concerns, perceptions, and attitudes [4–13], and discuss racial health disparities [14, 15] and health equity [16]. Furthermore, social media has played a crucial role in the dissemination of valuable information by universities, organizations, and governments to the public. Thus, social media platforms such as Twitter, Facebook, and Reddit are inundated with valuable information that presents a large data source that allows researchers opportunities to mine rich information using various methodologies. Increasingly, researchers are using natural language processing (NLP) and machine learning (ML) methods such as topic modeling and named-entity recognition (NER) to mine public health-related data.

Regarding health outcomes, preexisting social determinants of health (SDOH) have historically prevented marginalized racial and ethnic groups from equitable opportunities for physical, emotional, and socioeconomic health [17–19]. Public health crises tend to exacerbate SDOH and thereby their health outcomes even more. Researchers from multidisciplinary backgrounds have spent decades studying public health crises, health disparities, and the impacts of public health crises on the global public.

This study aims to assess social determinants of health issues of marginalized Black and Asian communities during the COVID-19 pandemic by leveraging NLP/ML techniques on race/ethnicity-specific spatiotemporal social media data. The social media platform of choice for this study is Reddit for the following reasons: (1) it is one of the most popular, user-created interest-driven social network platforms; (2) Reddit offers a diverse and inclusive space for individuals to share their stories, engage in dialog, and build solidarity with others facing similar challenges [20]; and (3) Reddit provides APIs to extract data from subreddits, which allowed us to explore location-specific publicly available data from Black and Asian communities.

The main contributions of this paper are summarized as follows.

- To the best of our knowledge, this is the first attempt to assess the social determinants of health factors of marginalized racial/ethnic US population groups that were disproportionately impacted by the COVID-19 pandemic by employing NLP/ML methods on Reddit social media data, specifically by using LDA/GSDMM-based topic modeling and custom named-entity recognition (NER).
- We built a cleaned corpus of African American/Asian-related posts from location subreddits of five highly populated racial/ethnic US cities by leveraging NLP/ML-based techniques. Subsequently, we compiled a uniquely comprehensive dataset of SDOH-related sentence samples.
- We identified that the COVID-19 pandemic exacerbated SDOH factors and increased the frequency of SDOH in Black and Asian communities by using our SDOH-based custom NER model.
- Our findings are consistent with non-NLP/ML-based results published in peer-reviewed publications concerning the impact of the COVID-19 pandemic on marginalized racial/ethnic population groups, particularly within the SDOH Social and Community Context (SCC) domain.

## Related Work

Recent research has increasingly demonstrated the effectiveness of using social media to study COVID-19. Some studies such as [4, 5] used NLP/ML techniques to analyze Reddit data for pandemic surveillance. The most utilized NLP/ML methods include LDA topic modeling and sentiment analysis [21, 22], whereas content analysis was the most utilized qualitative analysis method [21]. Twitter and Sina Weibo were the most utilized social media platforms. Tsao et al. [23] performed a scoping review of 81 peer-reviewed empirical studies relating to COVID-19 and social media: 45 of the 81 studies used the Twitter platform, 16 used Sina Weibo, and 4 used Reddit as data sources. In this study, we addressed the gap in the underexplored role of Reddit in COVID-19 surveillance.

Only a few studies utilized a combined NLP and qualitative approach for surveillance of COVID-19 using social media. Oyebode et al. [24] aimed to identify negative issues, positive opinions, and perceptions on social media. However, their approach did not explore the benefits of LDA/GSDMM topic modeling and custom NER in such analysis. This is important as topic modeling and custom NER can save a significant amount of time and effort when analyzing large text corpora.

Recent studies regarding COVID-19 and racial and ethnic US minority populations focused on negative consequences such as racism [25–27], structural COVID-19 disparities [28, 29], and anti-Asian discrimination [30, 31]. The primary US racial and ethnic groups the studies focused on were African American, Latino, Asian, Native Indian, and Pacific Islander communities. Moreover, these studies examined the disproportional impact of COVID-19 on US racial and ethnic minorities [26, 32, 33], addressed the needs and identified the disparities faced by these groups [34, 35], and examined their health risks and economic challenges due to the COVID-19 pandemic [36]. Collectively, these studies highlighted issues facing minority US populations such as an increased risk of COVID-19 infection due to certain underlying health conditions compared to Whites, difficulties accessing food and supplies, and disproportionate death rates. Though the knowledge gained from these studies is invaluable, many rely on small datasets and sample sizes, or tedious qualitative methods without the aid of efficient NLP/ML techniques. Nevertheless, these studies are an important step in the right direction, yet further review reveals a gap in understanding the social determinants of health issues of racial and ethnic US minority populations during the COVID-19 pandemic, specifically by applying NLP/ML to Reddit social media data.

Few studies explored the effects of COVID-19 using NLP on social media and/or race/ethnicity-specific data. For example, Su et al. [37] explored spatial–temporal factors and socioeconomic disparities that shaped US residents’ response to COVID-19. Adjei-Fremah et al. [38] found high variability in early transmission across the wards of D.C., which was driven by race/ethnic composition and SDOH. Odlum et al. [39] applied topic modeling and sentiment analysis techniques to tweets to inform designs of culturally sensitive interventions for COVID-19.

The remainder of this paper is outlined as follows. The “Methodology” section introduces techniques of dataset construction (data collection for topic modeling and custom NER, data preprocessing, training/evaluation dataset annotation), topic modeling, and NER (training, evaluation, and entity detection). The “Results and Discussions” section presents the results of topic modeling and NER followed by a discussion. The final sections provide the limitations and conclusions of this study.

## Methodology

The purpose of this section is to introduce the research methodology for this study regarding the social determinants of health issues of marginalized populations before and during the COVID-19 pandemic. Within the scope of this study, the racial/ethnic minorities we primarily focused on are Blacks and Asians. Due to a lack of specific race-related information, we did not differentiate between the various Asian races (i.e., Chinese, Japanese, Korean, etc.).

### Dataset Construction

The dataset construction involves data collection from Reddit social media platform, data preprocessing, and building a corpus for topic modeling. Additionally, a labeled custom NER training and evaluation dataset is constructed.

#### Data Collection for Topic Modeling

To extract and store the posts/comments, we employed Python scripts and used the following application programming interfaces (APIs): Python Reddit API Wrapper^4^ (PRAW) and Python Pushshift.io API Wrapper^5^ (PSAW). The search criteria for extracting posts and comments contained race-related keywords (Blacks OR African American OR Black people OR Black person; Asians OR Asian American OR Asian people OR Asian person) during two distinct periods: one prior to (03/01/2019–12/31/2019) and another during (03/01/2020–12/31/2020) the COVID-19 pandemic. We purposefully left the keyword search broad to eliminate bias in the dataset. Furthermore, topic modeling was performed to assess the core concepts/themes to shape the direction of the rest of the study. The locations of interest were five United States cities with large marginalized Black and Asian communities (New York City, NY; Los Angeles, CA; Chicago, IL; Philadelphia, PA; and Houston, TX).

**Fig. 1:**
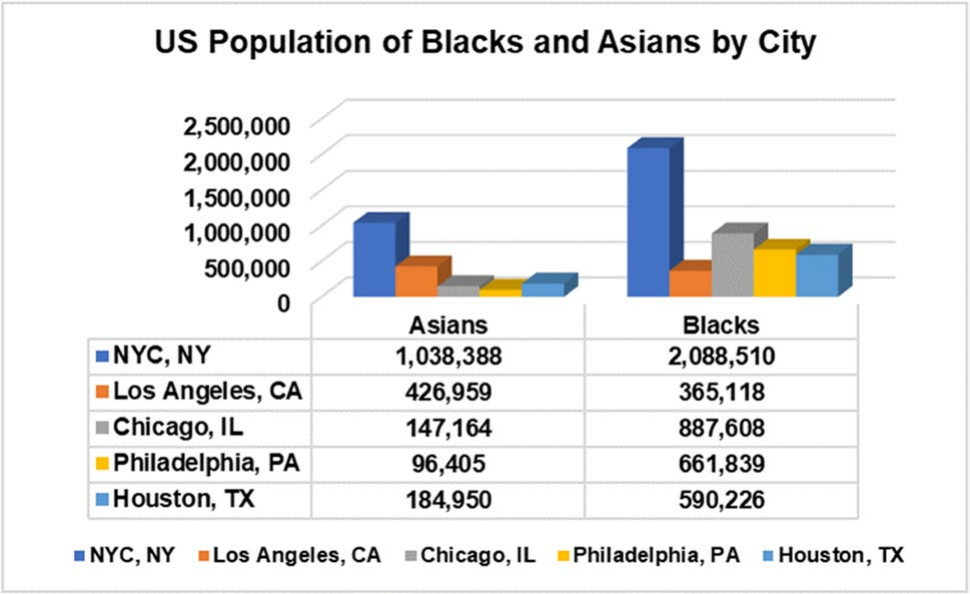
Population of US Blacks and Asians by location

**Fig. 2:**
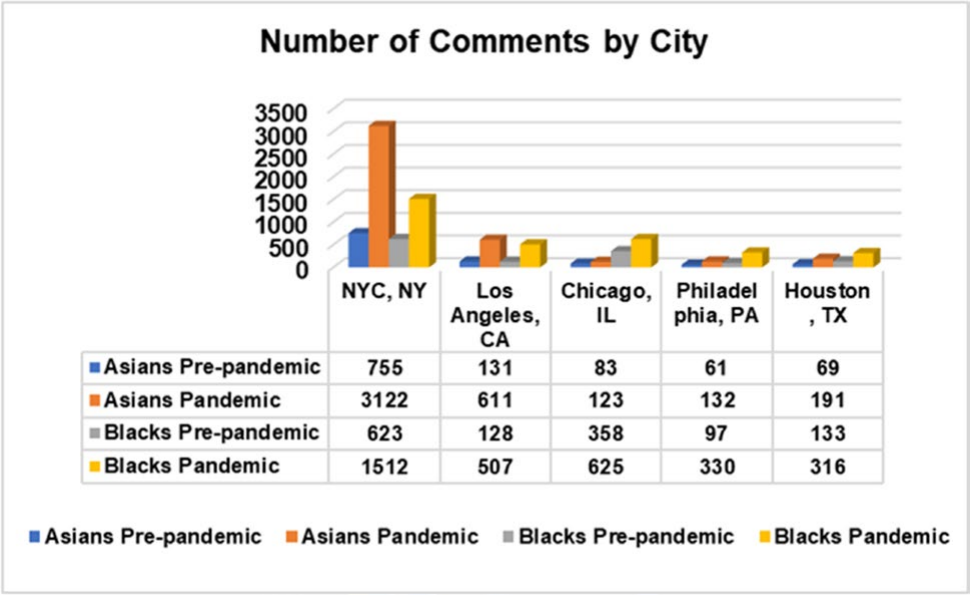
Number of extracted Reddit comments by location

**Fig. 3:**
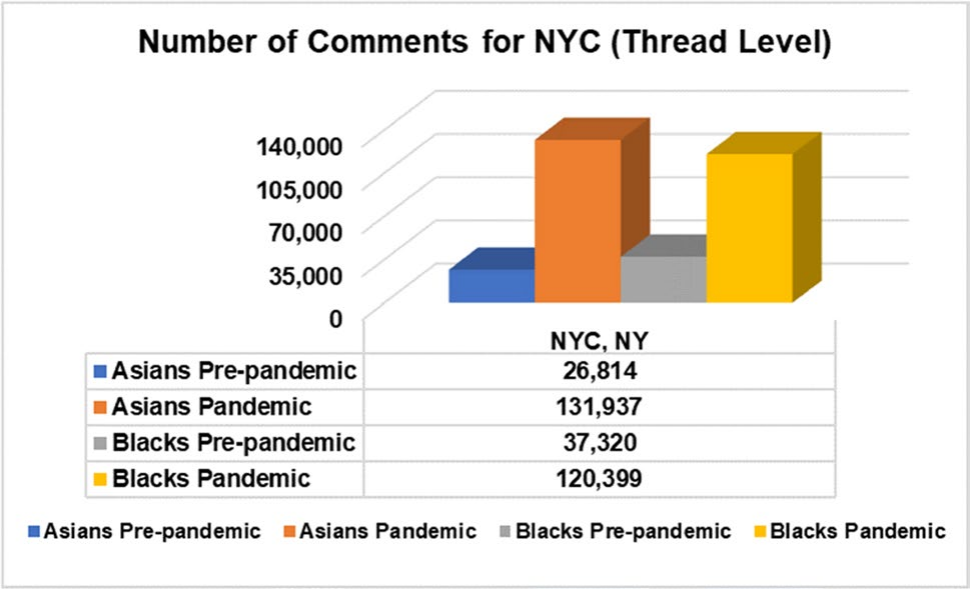
Total extracted conversation-level comments sourced in NYC

Reddit is one of the most popular, user-created interest-driven social network platforms. Additionally, Reddit has APIs that make data extraction easier, and it has been used for research on a variety of disease-related topics, including COVID-19 [4, 5] and monkeypox (Mpox) [40]. Subreddits are micro-communities within Reddit. The five subreddits we selected for this study were r/nyc with 866 K members, r/LosAngeles with 650 K members, r/chicago with 530 K members, r/philadelphia with 459 K members, and r/houston with 387 K members. We selected these location subreddits because these five locations are among the top 10 highest populations of African Americans and Asians in the US. The acquired data (with duplicates removed) were organized and stored according to target attributes in the appropriate text format. General statistics about our target population groups by location and African American (Blacks)/Asian datasets are shown in Figs. 1, 2, and 3.

#### Data Collection for NER

To provide sufficient data to train the custom NER model, we scraped additional Reddit data using PSAW with the keywords “Asians” and “Blacks” from January 1, 2018, through December 31, 2018. Using a modified approach to our topic modeling data collection procedure, we extracted comments containing the aforementioned keywords while omitting comments generated by bots, omitting comments containing less than 25 characters, and omitting comments containing the symbols “(” or “[.” We found that comments with those symbols mostly contained unintelligible strings. We then wrote the comments to a text file in UTF-8 text format which yielded 464,895 sentences.

#### Data Preprocessing

Data preprocessing^6^ is the process of converting raw data to a useful and efficient format for building models. In our study, data preprocessing served as a crucial step aimed at noise reduction and data normalization to achieve a cleaned state before conducting LDA (Latent Dirichlet Allocation) and GSDMM (Gibbs Sampling Dirichlet Multinomial Mixture) topic modeling. Our Python-based preprocessing procedures for topic modeling encompassed several key steps: decoding HTML Unicode strings to standard text format, lowercase conversion, tokenization, part-of-speech (POS) tagging, and lemmatization. Furthermore, preprocessing involved the removal of various elements such as URLs, usernames, excess whitespace, stop words, digits, punctuation, and non-ASCII characters. Notably, the terms “removed” and “deleted” were integrated into the stop words list to facilitate the exclusion of such terms, as comments were replaced with “removed” and “deleted” when thread-level comments became inaccessible on Reddit. Additionally, data preprocessing for custom named-entity recognition (NER) aimed to standardize sentence samples to align with the structure of formal and social media text. This process involved the formalization of proper nouns, condensation of repeating punctuation, substitution of whitespace between bigrams/trigrams with underscores, elimination of sentences containing non-relevant instances of the word “black(s)” (e.g., colors, paints, screen resolution), and conversion of alternative sentences to lowercase format. The latter was to ensure that we could train both uppercase and lowercase instances of proper nouns. Preprocessing source code is available upon request.^10^

#### Training/Evaluation Dataset Annotation

Using the extracted Reddit dataset in the “Data Collection for NER” section, we determined four social determinants of health-related named-entity categories and their respective keywords to use for sentence extraction following the Office of Disease Prevention and Health Promotion’s (ODPHP) Healthy People 2030 initiative. Additionally, we created a fifth category representing race and ethnicity. The categories are as follows: ECON (Economic Stability), EDU (Education), SCC (Social and Community Context), NBE (Neighborhood and Built Environment), and RETH (Race and Ethnicity). A few examples for each are as follows: ECON—poverty, employment, and wealth; EDU—education, high school, and college; SCC—racism, discrimination, and community; NBE—violent crime(s), neighborhoods, and crime(s); RETH—Asians, Blacks, and Hispanic. The reason we created the RETH category was to appraise the occurrences of Blacks and Asians for this study and to estimate the prevalence of the remaining races/ethnicities to determine if this dataset was sufficient for the follow-up study.

To build the annotated NER dataset from our raw sentence samples corpus, we used a dictionary lookup approach and extracted sentences from the text file containing case-insensitive NER keywords. For each keyword, we extracted 1000 sentences if the keyword sentence files contained 1000 or more samples. Otherwise, we extracted all sentences containing the NER keywords (if less than 1000 samples). As a result, we obtained 52,924 sentences (before removing duplicates) of which 70% was used for our training set (30,855 sentences) and 30% for evaluation (12,986 sentences). Next, we used Python scripts to prepare and label the training and evaluation datasets. We first tokenized each sentence and stored them in a column labeled “Words.” We then created an adjacent column labeled “Tags” and annotated each word with the appropriate SDOH-related NER category label. The training set yielded 843,101 words and the evaluation set yielded 355,649. The NER words in our annotated datasets were labeled using the BILUO^7^ annotation scheme. The composition of the resulting datasets is presented in Table 1.

**Table 1:** Number of labeled entity tags

Evaluation dataset		Training dataset	
Entity label	# of tags	Entity label	# of tags
ECON	2173	ECON	5072
EDU	1753	EDU	4353
NBE	1324	NBE	2791
SCC	6085	SCC	14,003
RETH	25,657	RETH	63,314

### Topic Modeling

A topic model is a statistical model that clusters documents into topics by discovering hidden semantic structures in a text corpus. Although there are other topic modeling approaches such as the author-topic model (ATM) [41], the most prevalent type of topic model utilized in natural language processing is Latent Dirichlet Allocation (LDA), which is a probabilistic topic modeling technique that necessitates the specification of a parameter *k* denoting the number of latent topics present within a given text corpus [42]. Initially, LDA randomly assigns each word in the corpus to one of the *k* topics, a process that is subsequently iteratively refined based on the distribution of each word across the *k* topics. Upon optimization, LDA incorporates the Term Frequency-Inverse Document Frequency (TF-IDF) metric, which assigns probabilities to words based on their frequency within documents, further adjusting these probabilities according to their overall frequency across the corpus. This iterative refinement process continues until reaching a user-defined convergence threshold or until iterations cease to substantially impact the probability assignments to words within the text corpus.

Comparably, Gibbs Sampling Dirichlet Multinomial Mixture (GSDMM) [43] is a topic modeling algorithm designed to cluster text data into coherent topics. GSDMM operates by iteratively assigning each document to a topic based on the frequency of words within that document and the prevalence of topics across the corpus. Initially, GSDMM randomly assigns documents to topics and calculates the likelihood of each document belonging to each topic. It then iteratively updates these assignments based on statistical inference, adjusting topic assignments to maximize coherence within clusters. This iterative process continues until convergence is reached, resulting in the identification of coherent topic clusters within the text corpus.

In this study, we implemented Java-based LDA topic models using MALLET, and also Python-based comment-level topic models using GSDMM to assess the SDOH factors of African and Asian Americans before and during the COVID-19 pandemic. We assigned *k* = 7 topics for the LDA comment-level topic models, seven clusters for GSDMM (for consistency), and *k* = 16 for the LDA thread-level topic models. Additionally, we obtained interactive pyLDAvis visualization files for the LDA topic models and the word clouds (image files) of the top 20 frequent keywords for the GSDMM topic models to aid in topic/theme interpretation.

#### Design and Implementation

We implemented the first comment-level LDA topic models using MALLET, which is a Java-based toolset for NLP tasks, such as document classification, clustering, and topic modeling. Similarly, we implemented comment-level LDA topic models using Gensim, a Python library for topic modeling, similarity retrieval, and document indexing. First, we imported and processed our cleaned dataset to generate bigrams, create a vocabulary dictionary, and construct a Term Document Frequency data corpus. Next, we passed the processed data to the respective LDA modules to generate various topic models using the recommended optimization parameters. For each comment-level dataset, we generated four topic models (each with *k* = 4, 7, 10, and 13 topics) to assess their corresponding coherence scores to assist in determining the optimal model. The coherence scores exhibited variability across the datasets, prompting the calculation of averages based on the number of topics associated with each coherence score. The resulting average number of topics for Gensim was determined to be 8.25, while for MALLET, it was calculated to be 7 (combined Gensim/MALLET average is 7.6 topics). Additionally, the collective average coherence score across all LDA comment-level datasets was calculated to be 0.38. Moreover, we visually inspected various random topics to further assess the optimal number of topics for the comment-level datasets and concluded *k* = 7 topics.

Similarly, for each thread-level dataset, we generated six topic models (each with *k* = 4, 7, 10, 13, 16, and 19 topics). The fluctuation in coherence scores exhibited a notably reduced degree of variability compared to that observed in the comment-level datasets, which can likely be attributed to the similarity in sizes between the thread-level datasets. In most cases, *k* = 16 topics yielded the top two highest coherence scores with an average score of 0.43 for *k* = 16 topics across all thread-level datasets. Consequently, we concluded that *k* = 16 was the optimal number of topics for the thread-level LDA-based topic models. By design, GSDMM is optimized for datasets containing short text and assumes each document (comment) is about only one topic, thus we exclusively evaluated LDA-based topic models for thread-level datasets as they contain entire conversations about diverse topics.

In implementing the remaining comment-level topic models utilizing the GSDMM approach, several procedural steps were followed. Initially, the cleaned dataset was imported and processed to facilitate the creation of a vocabulary dictionary, aimed at capturing the unique terms present within the dataset. Subsequently, we filtered out extreme cases from the dictionary, ensuring completeness of the vocabulary. Additionally, a Term Document Frequency (TDF) data corpus was constructed, enabling the quantification of term occurrences across the dataset.

Following these preparatory steps, the processed data were passed to GSDMM’s topic modeling module, where various topic models were generated utilizing the optimization parameters recommended by the algorithm. Additionally, seven clusters, each containing twenty keywords, were assigned for each comment-level dataset and saved to a text file for further processing. Through this systematic approach, the GSDMM method was effectively employed to derive topic models from the comment-level dataset, facilitating the evaluation and analysis of thematic patterns within the race/ethnicity-related data corpus.

### Named-Entity Recognition (NER)

NER is a sub-task of Information Extraction (IE) which aims to classify certain named-entities found within an unstructured body of a text corpus [44]. Entities found using the NER task are typically classified into pre-defined categories such as person, location, organization, medical codes, and disease names. NER models are required to be evaluated to check the validity of their performance by comparing the outputs typically against human-annotated tags. The comparisons are generally quantified simultaneously by attempting to correctly recognize a detected instance’s boundary and its entity type. Consequently, accurate predictions using the performance metrics are assessed.^8^ The custom NER model trained in this study is based on spaCy’s multi-task convolutional neural network (CNN) which was trained using the OntoNotes^9^ corpus, and contains GloVe vectors [45] that were trained on Common Crawl.

#### Training and Evaluation

To train our custom SDOH-based NER model, we first created labels from our predetermined NER categories, provided a model name, and created a NER pipeline. We followed spaCy’s recommendations for fine-tuning the following hyperparameters: training iterations, batch size, and dropout rate. A batch size begins at a user-defined minimum and each batch increases until it reaches a user-defined maximum threshold. Dropout is a stochastic regularization technique that aims to reduce overfitting in neural networks by temporarily removing neurons during training [46]. Our custom NER model was trained with 50 iterations, a batch size from 1 to 16, and a dropout rate of 0.35. Upon training our custom NER model, we evaluated it by loading our annotated evaluation dataset in spaCy Scorer and achieved the following performance metrics: precision = 0.92, recall = 0.99, and F1-score = 0.95.

#### Entity Detection

To detect our named-entities, we performed a custom NER task on our cleaned Reddit corpus. We then extracted and stored the named-entities and their corresponding tags. Finally, we processed each document from our Reddit corpus individually to the document parser and appended the detected named-entities and tags to our respective tab-separated values files.

## Results and Discussions

**Fig. 4:**
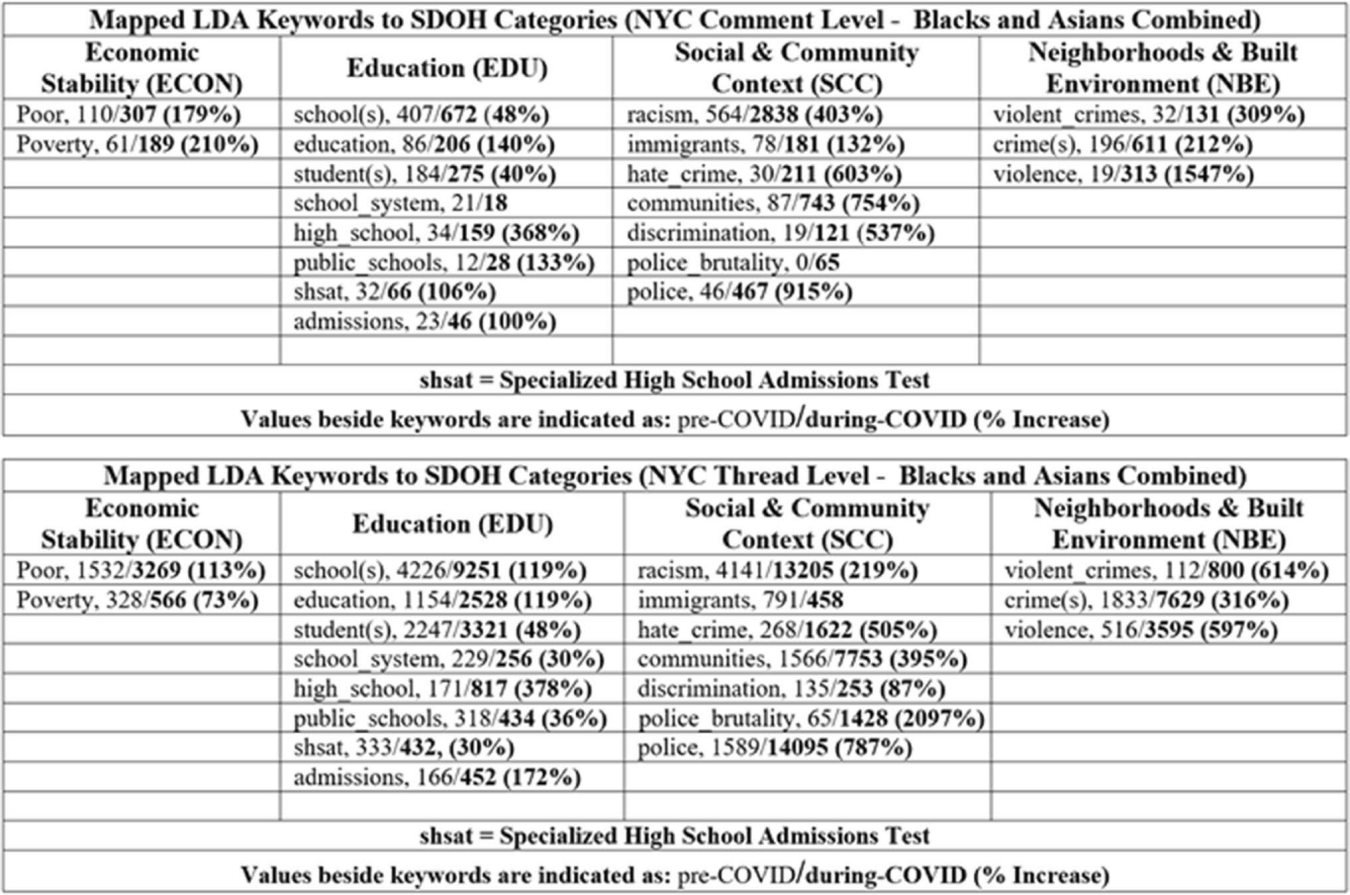
Mapped LDA keywords to SDOH categories. Values are indicated as pre-COVID/during-COVID (% increase)

This section outlines the results of our study to include general statistics of our African American/Asian datasets, topic modeling and custom NER analysis, and discussions (Fig. 4). Our datasets are available upon request.^10^

### What SDOH Categories are Discussed?

The topics discovered through LDA methods were analyzed and interpreted using the top 30 prominent keywords displayed in the interactive pyLDAvis visualization files. The topics discovered through GSDMM methods were interpreted using the top 20 prominent keywords in the word cloud image files representing each topic. The primary purpose for the topic modeling phase of this study was to glean the primary topics and themes from our race/ethnicity-related dataset and to shape the next phase of our study (custom NER).

#### Topic Modeling Analysis and Findings

**Fig. 5:**
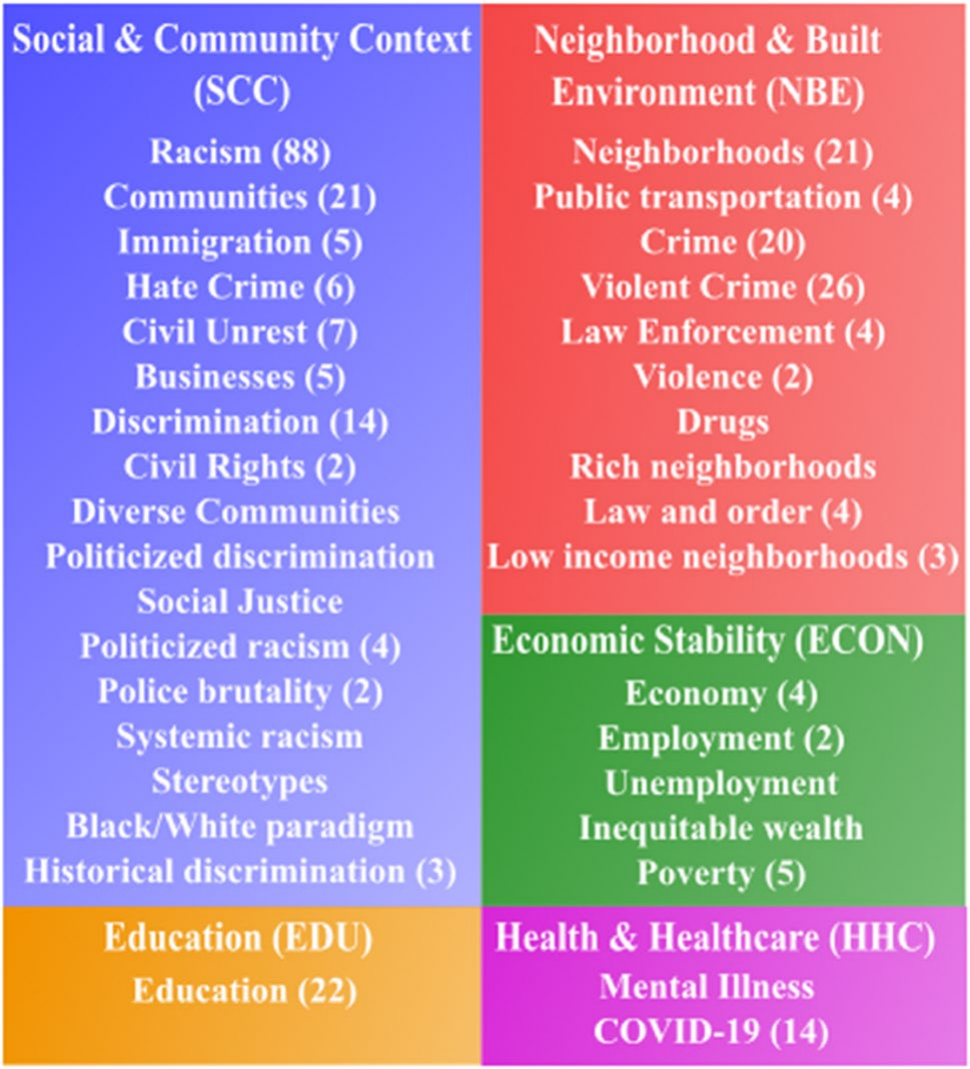
Mapped topics to SDOH categories. Values indicate the frequency of an occurred topic throughout all datasets

Initially, we established five overarching categories aligned with the SDOH domains outlined by the Healthy People 2030 initiative.^11^ These categories were further delineated into subcategories (i.e., Social and Community Context → civic participation, discrimination, incarceration, social cohesion, racism, racialized legal status/immigration, etc.), providing a comprehensive framework for classifying SDOH-related topics. Next, we compiled topics generated from both LDA and GSDMM modeling analyses conducted at the comment-level across five regions, along with LDA modeling results specific to New York City (NYC) at the thread-level. By consolidating these topic datasets, we accumulated a pool of interpreted topics and their corresponding frequencies. Subsequently, we meticulously mapped these topics to the predefined SDOH categories as depicted in Fig. 5. Additionally, to further validate our mapping methodology, we conducted a sampling process wherein LDA keywords from NYC thread-level datasets were systematically mapped similarly, as illustrated in Fig. 4. This comprehensive mapping strategy ensured that our analysis accurately captured and categorized relevant SDOH information embedded within the LDA and GSDMM-modeled topics, thereby enhancing the reproducibility of our findings.

Initially, we included the SDOH category Health and Healthcare (HHC); however, we omitted it in subsequent analyses due to the insufficient number of non-COVID-19 health-related topics mapped to that category. However, the COVID-19-related topics validated that our data collected during the pandemic was a sufficient proxy, without influencing the data by explicitly including COVID-19-related keywords with our race/ethnicity keyword search during data collection.

#### Interpretation and Implications of Topic Modeling Results

The observed increase in the frequency of comments related to African Americans and Asians during the pandemic demonstrates how public health crises can significantly influence online discourse on Reddit. The heightened discussions about racial and ethnic communities amidst the pandemic further revealed the pressing need for a better understanding of SDOH issues experienced by these marginalized population groups. By leveraging efficient topic modeling techniques, we identified and characterized 35 SDOH-related topics, representing a myriad of factors influencing public health outcomes and disparities. These topics served as the foundation for constructing a custom NER model, enabling more precise identification and extraction of SDOH-related information from our racial/ethnic-based data corpus.

Moreover, the surge in the frequency of topics and associated keywords during the pandemic period suggests a heightened awareness and discussion surrounding public health-related issues within these communities. This finding carries significant implications for public health interventions and policy initiatives aimed at addressing health disparities among African American and Asian populations. By revealing the specific SDOH factors discussed before and during the pandemic, our research provides valuable insights for policymakers, healthcare practitioners, and community stakeholders to develop interventions and allocate resources more effectively. Furthermore, these findings highlight the importance of monitoring and responding to emerging trends in online discourse as a means of understanding public perceptions and priorities related to public health and social issues. Overall, our research contributes to a deeper understanding of the intersection between online discourse, public health, and social determinants of health, with implications for informing evidence-based interventions and strategies to promote health equity and well-being among marginalized racial/ethnic populations.

### What Social Determinants of Health Factors are Discussed?

To further assess the SDOH factors, we performed custom NER using our trained SDOH-based custom NER model on the cleaned race/ethnicity dataset. We recorded the detected named-entities in tables grouped by the targeted locations, the specified time periods, and the targeted Black and Asian communities. We analyzed SDOH and race/ethnicity-based entities from all the spatiotemporal comment-level datasets and the thread-level datasets from NYC. Moreover, we calculated the percent increase for both periods for the targeted population groups to assess if the COVID-19 pandemic exacerbated SDOH factors and/or increased the frequency of the mentioning of racial/ethnic population groups.

#### Custom NER Analysis and Findings

**Fig. 6:**
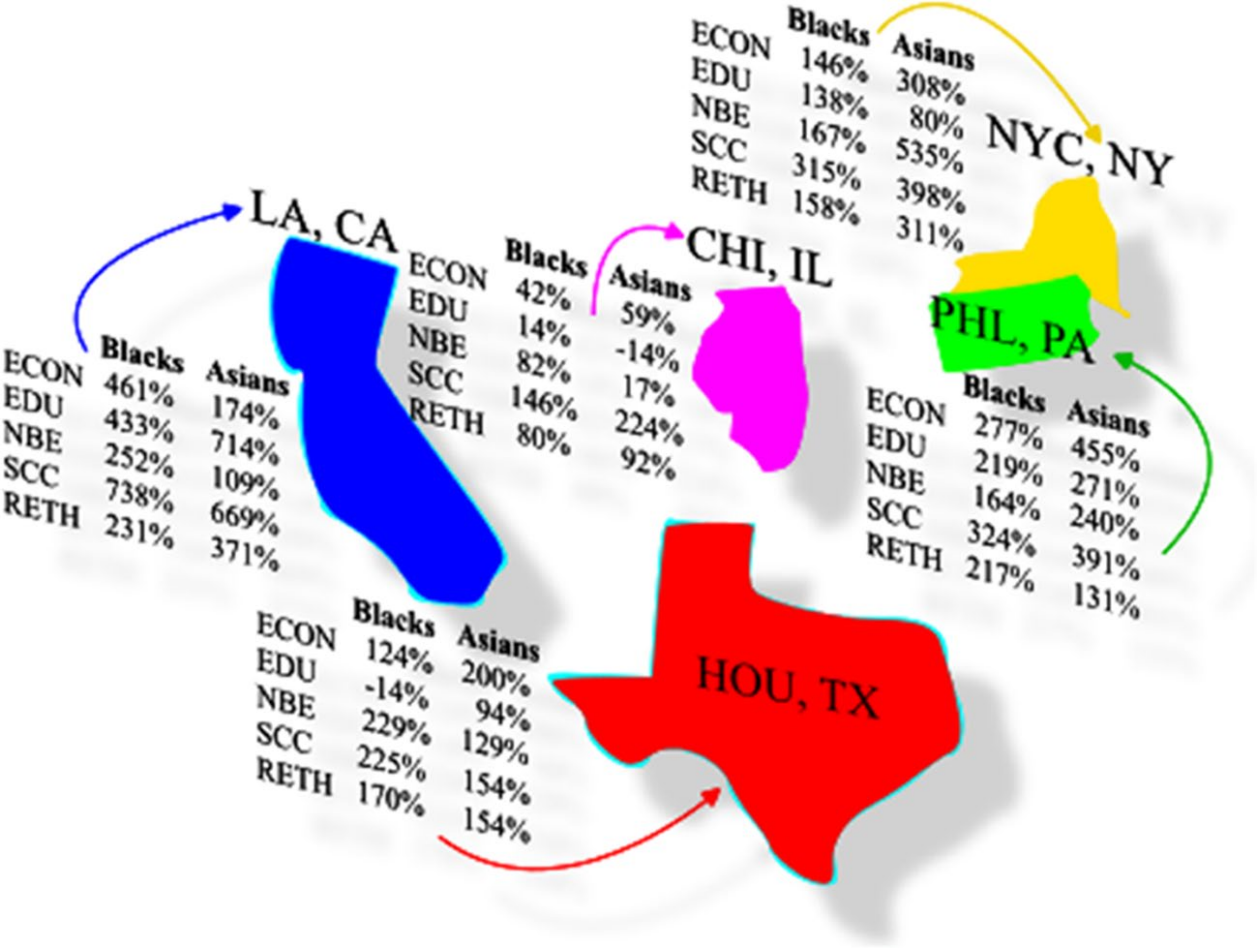
Percent increase/decrease per custom NER category per location during COVID-19 pandemic

Regarding the comment-level datasets from the subreddits r/nyc, r/LosAngeles, r/chicago, r/philadelphia, and r/houston, each category saw a significant percent increase (Fig. 6) except EDU which incurred a 14% decrease for Asians in Chicago and Blacks in Houston. The highest percent increase occurred in LA for Blacks in the SCC category (738%). The highest percent increase for Asians also occurred in LA, however in the EDU category (714%). In terms of average, LA also had the largest percent increase across all categories (423% Blacks, 410% Asians). Regarding individual categories across all locations and population groups: SCC had the highest average percent increase from the pre-pandemic period to the pandemic period (366%), followed by ECON (226%), RETH (198%), NBE (192%), and EDU (185%). When comparing Blacks and Asians separately across all locations, the average percent increases are as follows: Blacks—SCC (350%), ECON (210%), NBE (179%), RETH (171%), and EDU (158%); Asians—SCC (367%), ECON (239%), EDU (229%), RETH (212%), and NBE (206%).

**Fig. 7:**
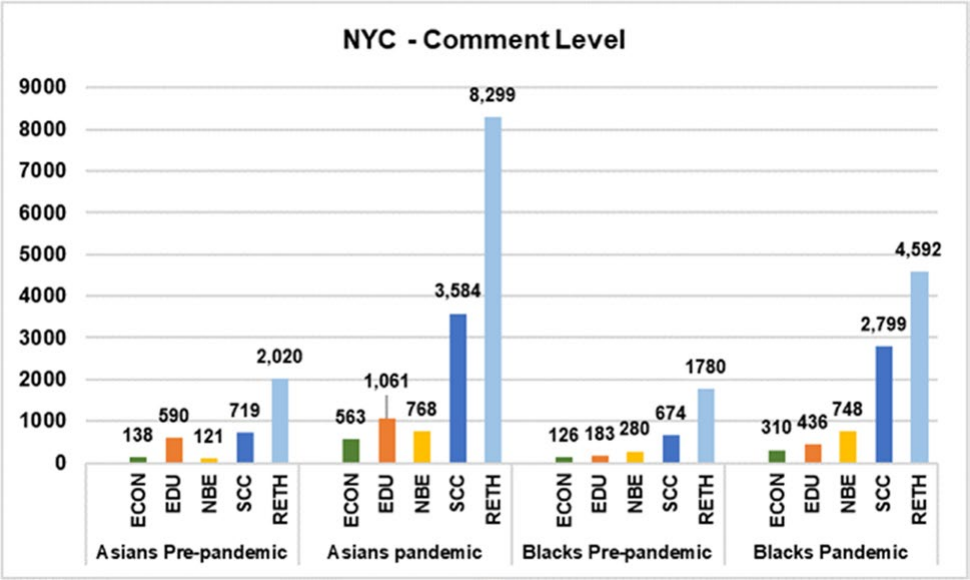
Number of detected entities prior and during COVID-19 per category (NYC—comment-level)

**Fig. 8:**
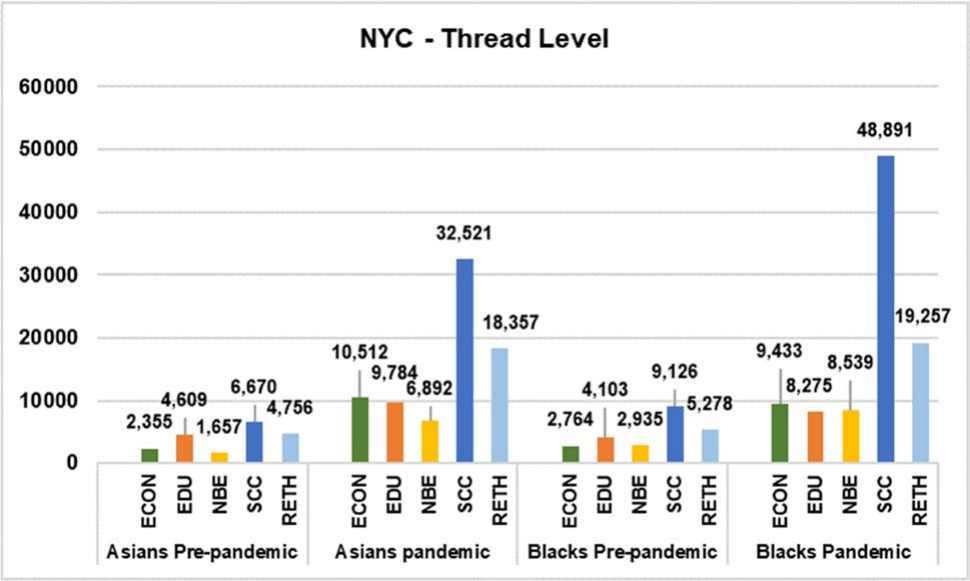
Number of detected entities before and during COVID-19 per category (NYC—conversation thread-level)

The remaining custom NER analysis for this study pertains to NYC comment and thread-level datasets, considering that NYC has the highest representative sample populations of US Blacks and Asians. To determine if the pandemic exacerbated SDOH issues affecting Blacks and Asians, we analyzed the detected named-entities for each category during both periods, showing a significant increase across all categories (Figs. 7 and 8). We found that Asians experienced the highest percent increase in the NBE category (535%) and Blacks in the SCC category (315%) from the NYC comment-level dataset. Regarding the NYC thread-level dataset, both Asians and Blacks experienced the highest percent increase in the SCC category (388% and 436%, respectively).

To further characterize the SDOH factors impacting Blacks and Asians, we analyzed the most prevalent underlying key issues from each SDOH category in addition to the most prevalent mentioned races/ethnicities (Tables 2 and 3). If an SDOH entity was detected at least one hundred times (during the pandemic period), we included them in the tables. Nearly all SDOH factors for each category significantly increased from the pre-pandemic period to the pandemic period. Consistent with the named-entity category analysis, the highest percentage increase for individual SDOH factor analysis was also observed in the SCC category for both Blacks and Asians. Regarding one of the most prominent SCC factors, police brutality had the highest percent increase for both Blacks and Asians in the comment and thread-level datasets (Blacks—6000%, Asians—2100%; Blacks—1982%, Asians—2155%, respectively). Civic participation (i.e., protests—Black Lives Matter, volunteering, etc.), a key issue in the SCC domain, had a sharp increase despite COVID-19 being a global public health crisis. The increase in Black Lives Matter (BLM)-related protests is largely in response to the surge in police brutality and social injustice observed during the pandemic [47, 48].

#### Interpretation and Implications of NER Results

Generally, this study found that Blacks and Asians were most impacted by factors within the SDOH domain Social and Community Context (SCC). The SCC domain reflects a number of key issues that make up the underlying factors affecting their socioeconomic health including civic participation, discrimination, incarceration, racism, racialized legal status/immigration, etc. Our findings are largely consistent with results derived by other methodologies published in peer-reviewed publications concerning the impact of the COVID-19 pandemic on marginalized racial/ethnic population groups [49–52], specifically within the SDOH SCC domain. These studies derived their conclusions without leveraging social media and NLP/ML methods, thus highlighting the viability of using our approach for similar research aims. Moreover, these findings can inform targeted interventions and policy initiatives aimed at addressing the root causes of health inequities among racially and ethnically diverse populations. By understanding the specific challenges faced by these groups, stakeholders can develop more effective strategies to promote health equity and social justice in diverse communities. Furthermore, this research highlights the importance of ongoing monitoring and surveillance of SDOH factors to track trends over time and inform evidence-based decision-making to mitigate public health inequities.

### Future Work

In our study, topic modeling revealed that the COVID-19 pandemic affected the SDOH of racial and ethnic minority groups. Further analysis of their SDOH issues using the custom NER method found that racial and ethnic minority communities had the highest average percent increase in the SCC domain during the pandemic. The discussions about SDOH issues of marginalized populations drastically increased during the pandemic. To further examine the prevalent SDOH-related themes and nuanced discussions about all affected marginalized racial/ethnic groups identified in this study, a thorough thematic discourse analysis in a follow-up study could be conducted using our comprehensive dataset.

## Limitations

As with the majority of studies, this study has a few limitations. First, we had to normalize some of the data to calculate the percent increases/decreases. There were two instances of normalizations in our African American dataset and four instances in our Asian dataset (Tables 2 and 3). Thus, if no named-entities were detected prior to the pandemic, we normalized the “0” values with “1” prior to calculating the percent increase/decrease during the pandemic. Second, although we used the Asian-based keywords for data extraction, our NER model recognized specific ethnic subgroups such as Chinese, Japanese, Korean, Vietnamese, Filipino, Indian, Pakistani, and others. However, not all representations of these subgroups were adequate in our data for meaningful analysis and therefore not represented in Tables 2 and 3. We recognize this limitation of not having a complete subgroup analysis for Asians which can be addressed in a future study. Finally, generalizations alone may not be enough to infer how the pandemic affected the SDOH issues of additional racial and ethnic groups detected in this study (non-Blacks and non-Asians). For example, there were 1469 occurrences of Hispanic/Latino in the African American COVID-19 dataset; however, without a deeper analysis of the comments that explicitly contain Hispanic/Latino keywords, accurate inferences cannot be made. Thus, this limitation can be addressed by conducting a thorough thematic discourse analysis on our racial/ethnic datasets in a follow-up study.

## Conclusion

As evident from prior public health crises, the COVID-19 pandemic may disproportionately affect marginalized racial/ethnic population groups. Currently, in the United States, 40% of the population identifies as racial or ethnic minorities, of which 13.6% are Black and 6.3% are Asian. Therefore, a significant percentage of the US population’s health can be disproportionately impacted by the COVID-19 pandemic. Thus, it is critically important to assess the impact of COVID-19 on the social determinants of health of Black and Asian communities so that policymakers and service providers can conclusively formulate responses and policies to prevent similar impacts from future public health crises. With social media becoming the platform of choice for many people to express their perceptions, attitudes, and concerns regarding the COVID-19 pandemic, it presents an opportunity for researchers to employ various methodologies such as NLP/ML to understand the impacts of the pandemic on affected groups. Our study has successfully demonstrated the feasibility of understanding the impact of the COVID-19 pandemic by applying NLP/ML to spatiotemporal-specific social media data, specifically with LDA/GSDMM topic modeling and custom NER. Moreover, our review of the literature revealed a gap in understanding the impacts of the pandemic on marginalized racial and ethnic US population groups. Thus, our study shifted the focus to understanding the impact of the COVID-19 pandemic on the SDOH factors of marginalized Black and Asian communities. The results of our study corroborate those of related studies, which further sheds light on the need to address the social determinants of race/ethnic minorities during a public health crisis.

## Data Availability

Available upon request.

## Appendix

**Table 2:** Total number of detected entities by category with % increases/decreases from pre-pandemic to pandemic (Blacks)

New York City, NY				
	**Comment-level**		**Thread-level**	
**Entity label**	**Blacks pre-pandemic**	**Blacks pandemic**	**Blacks pre-pandemic**	**Blacks pandemic**
**ECON**	Job(s) **(24)**	Job(s) **(90) *****275%***	Job(s) **(942)**	Job(s) **(3508) *****272%***
	Business(es) **(12)**	Business(es) **(61) *****408%***	Business(es) **(407)**	Business(es) **(2436) *****499%***
	Homeless(ness) **(8)**	Homeless(ness) **(10) *****25%***	Homeless(ness) **(569)**	Homeless(ness) **(877) *****54%***
	Wealth(y) **(12)**	Wealth(y) **(51) *****325%***	Wealth(y) **(274)**	Wealth(y) **(726) *****165%***
	Poverty **(33)**	Poverty **(68) *****106%***	Poverty **(207)**	Poverty **(706) *****241%***
	Unemployment **(3)**	Unemployment **(2) − *****33%***	Unemployment **(49)**	Unemployment **(676) *****1280%***
	Welfare **(26)**	Welfare **(19) − *****27%***	Welfare **(122)**	Welfare **(220) *****80%***
**EDU**	School **(83)**	School **(221) *****166%***	School **(2303**)	School **(4413) *****92%***
	Education **(45)**	Education **(114) *****153%***	Education **(781)**	Education **(1867) *****139%***
	College **(11)**	College **(36) *****227%***	College **(378)**	College **(809) *****114%***
	High school **(23)**	High school **(34) *****48%***	High school **(364)**	High school **(684) *****88%***
	University **(9)**	University **(11) *****22%***	University **(121)**	University **(205) *****69%***
**NBE**	Crime(s) **(177)**	Crime(s) **(492) *****178%***	Crime(s) **(1944)**	Crime(s) **(5551) *****186%***
	Neighborhood(s) **(55)**	Neighborhood(s) **(135) *****145%***	Neighborhood(s) **(798)**	Neighborhood(s) **(2412) *****202%***
	Violent crime(s) **(48)**	Violent crime(s) **(120) *****150%***	Violent crime(s) **(187)**	Violent crime(s) **(556) *****197%***
**SCC**	Police/cop(s) **(87)**	Police/cop(s) **(724) *****732%***	Police/cop(s) **(2390)**	Police/cop(s) **(17,605) *****637%***
	Protest(s) **(7)**	Protest(s) **(159) *****2171%***	Protest(s) **(251)**	Protest(s) **(5799) *****2210%***
	Racism **(274)**	Racism **(887) *****224%***	Racism **(2594)**	Racism **(9255) *****257%***
	Community **(90)**	Community **(4296) *****4673%***	Community **(996)**	Community **(4326) *****334%***
	Arrest(ed) **(56)**	Arrest(ed) **(118) *****111%***	Arrest(ed) **(641)**	Arrest(ed) **(2437) *****280%***
	Black Lives Matter **(7)**	Black Lives Matter **(153) *****2086%***	Black Lives Matter **(84)**	Black Lives Matter **(2165) *****2477%***
	Protestor(s) **(1)**	Protestor(s) **(50) *****4900%***	Protestor(s) **(355)**	Protestor(s) **(2650) *****2280%***
	Immigration **(50)**	Immigration **(110) *****120%***	Immigration **(1086)**	Immigration **(1257) *****16%***
	Police brutality **(0)**	Police brutality **(61) *****6000%***	Police brutality **(39)**	Police brutality **(812) *****1982%***
	Hate crime **(14)**	Hate crime **(85) *****507%***	Hate crime **(309)**	Hate crime **(704) *****128%***
	White supremacy **(18)**	White supremacy **(55) *****206%***	White supremacy **(176)**	White supremacy **(591) *****236%***
	Discrimination **(35)**	Discrimination **(91) *****160%***	Discrimination **(236)**	Discrimination **(610) *****158%***
	Stereotype **(21)**	Stereotype(s) **(31) *****48%***	Stereotype(s) **(323)**	Stereotype(s) **(323) *****0%***
	Segregation **(17)**	Segregation **(26) *****53%***	Segregation **(136)**	Segregation **(324) *****138%***
**RETH**	Black people **(112)**	Black people **(751) *****571%***	Black people **(724)**	Black people **(2996) *****314%***
	Asians **(130)**	Asians **(584) *****349%***	Asians **(554)**	Asians **(2983) *****438%***
	Chinese **(46)**	Chinese **(136) *****196%***	Chinese **(338)**	Chinese **(1928) *****470%***
	White people **(80)**	White people **(198) *****148%***	White people **(676)**	White people **(1745) *****158%***
	Blacks **(763)**	Blacks **(1910) *****150%***	Blacks **(570)**	Blacks **(1499) *****163%***
	Hispanic **(198)**	Hispanic **(225) *****14%***	Hispanic **(524)**	Hispanic **(804) *****53%***
	Whites **(201)**	Whites **(405) *****101%***	Whites **(362)**	Whites **(731) *****102%***
	Asian American **(8)**	Asian American **(53) *****563%***	Asian American **(82)**	Asian American **(670) *****717%***
	Latino **(79)**	Latino **(153) *****94%***	Latino **(181)**	Latino **(555) *****207%***
	Person(s) of Color **(2)**	Person(s) of Color **(33) *****1550%***	Person(s) of Color **(110)**	Person(s) of Color **(511) *****365%***
	African American **(35)**	African American **(138) *****294%***	African American **(156)**	African American **(658) *****322%***
	Asian people **(10)**	Asian people **(61) *****510%***	Asian people **(56)**	Asian people **(433) *****673%***
	Korean **(8)**	Korean **(15) *****88%***	Korean **(24)**	Korean **(294) *****1125%***
	Indian **(16)**	Indian **(23) *****44%***	Indian **(72)**	Indian **(266) *****269%***
	Black man (men) **(17)**	Black man (men) **(56) *****229%***	Black man (men) **(94)**	Black man (men) **(486) *****417%***
	Japanese **(0)**	Japanese **(20) *****1900%***	Japanese **(19)**	Japanese **(206) *****984%***
	Native American **(6)**	Native American **(10) *****67%***	Native American **(47)**	Native American **(173) *****268%***
	White man** (3)**	White man **(22) *****633%***	White man** (29)**	White man **(145) *****400%***
	Black woman **(11)**	Black woman **(10) − *****9%***	Black woman **(51)**	Black woman **(140) *****175%***
	White woman **(6)**	White woman **(12) *****100%***	White woman **(45)**	White woman **(122) *****171%***
	Mexican **(6)**	Mexican **(13) *****117%***	Mexican **(61)**	Mexican **(116) *****90%***
	LatinX **(1)**	LatinX **(14) *****1300%***	LatinX **(13)**	LatinX **(110) *****746%***

**Table 3:** Total number of detected entities by category with % increases/decreases from pre-pandemic to pandemic (Asians)

New York City, NY				
	**Comment-level**		**Thread-level**	
**Entity label**	**Asians pre-pandemic**	**Asians pandemic**	**Asians pre-pandemic**	**Asians pandemic**
**ECON**	Job(s) **(32)**	Job(s) **(121) *****278%***	Job(s) **(749)**	Job(s) **(3450) *****361%***
	Business(es) **(14)**	Business(es) **(161) *****1050%***	Business(es) **(356)**	Business(es) **(3120) *****776%***
	Homeless(ness) **(10)**	Homeless(ness) **(20) *****100%***	Homeless(ness) **(536)**	Homeless(ness) **(937) *****75%***
	Unemployment **(0)**	Unemployment **(9) *****800%***	Unemployment **(11)**	Unemployment **(928) *****8336%***
	Poverty **(53)**	Poverty **(176) *****232%***	Poverty **(194)**	Poverty **(687) *****254%***
	Wealth(y) **(25)**	Wealth(y) **(59) *****136%***	Wealth(y) **(318)**	Wealth(y) **(729) *****129%***
	Welfare **(3)**	Welfare **(8) *****167%***	Welfare **(85)**	Welfare **(195) *****129%***
	Employment **(0)**	Employment **(6) *****500%***	Employment **(24)**	Employment **(128) *****433%***
**EDU**	School **(341)**	School **(572) *****68%***	School **(2847)**	School **(5749) *****102%***
	Education **(97)**	Education **(243) *****151%***	Education **(850)**	Education **(1921) *****126%***
	College **(39)**	College **(66) *****69%***	College **(282)**	College **(888) *****215%***
	High school **(94)**	High school **(134) *****43%***	High school **(494)**	High school **(779) *****58%***
	University **(12)**	University **(33) *****175%***	University **(83)**	University **(275) *****231%***
**NBE**	Crime(s) **(67)**	Crime(s) **(543) *****710%***	Crime(s) **(1003)**	Crime(s) **(4331) *****332%***
	Neighborhood(s) **(40)**	Neighborhood(s) **(156) *****290%***	Neighborhood(s) **(572)**	Neighborhood(s) **(2151) *****276%***
	Violent crime(s) **(14)**	Violent crime(s) **(68) *****386%***	Violent crime(s) **(81)**	Violent crime(s) **(402) *****396%***
**SCC**	Police/cop(s) **(32)**	Police/cop(s) **(171) *****434%***	Police/cop(s) **(1478)**	Police/cop(s) **(9005) *****509%***
	Racism **(316)**	Racism **(1997) *****532%***	Racism **(1901)**	Racism **(8306) *****337%***
	Protest(s) **(12)**	Protest(s) **(98) *****717%***	Protest(s) **(154)**	Protest(s) **(3573) *****2220%***
	Community **(71)**	Community **(429) *****504%***	Community **(706)**	Community **(3494) *****395%***
	Black Lives Matter **(3)**	Black Lives Matter **(135) *****4400%***	Black Lives Matter **(25)**	Black Lives Matter **(1585) *****6240%***
	Arrest(ed) **(19)**	Arrest(ed) **(35) *****84%***	Arrest(ed) **(351)**	Arrest(ed) **(1242) *****254%***
	Immigration **(91)**	Immigration **(201) *****121%***	Immigration **(1281)**	Immigration **(1055) − *****18%***
	Protestor(s) **(18)**	Protestor(s) **(115) *****539%***	Protestor(s) **(234)**	Protestor(s) **(1484) *****534%***
	Hate crime **(26)**	Hate crime **(221) *****750%***	Hate crime **(229)**	Hate crime **(780) *****241%***
	Police brutality** (0)**	Police brutality **(22) *****2100%***	Police brutality** (20)**	Police brutality **(451) *****2155%***
	Discrimination (**58)**	Discrimination (**240) *****314%***	Discrimination **(191)**	Discrimination (**611) *****220%***
	Stereotype(s) **(37)**	Stereotype(s) **(97) *****162%***	Stereotype(s) **(94)**	Stereotype(s) **(340) *****262%***
	White supremacy **(25)**	White supremacy **(61) *****144%***	White supremacy **(109)**	White supremacy **(395) *****262%***
	Segregation **(32)**	Segregation **(28) − *****13%***	Segregation **(132)**	Segregation **(291) *****120%***
**RETH**	Asians** (983)**	Asians **(4333) *****341%***	Asians** (912)**	Asians **(3824) *****319%***
	Chinese **(134)**	Chinese **(660) *****393%***	Chinese **(582)**	Chinese **(2676) *****360%***
	Black people **(73)**	Black people **(549) *****652%***	Black people **(358)**	Black people **(2234) *****524%***
	White people **(70)**	White people **(250) *****257%***	White people **(431)**	White people **(1349) *****213%***
	Blacks **(110)**	Blacks **(491) *****346%***	Blacks **(306)**	Blacks **(917) *****200%***
	Asian American **(78)**	Asian American **(308) *****295%***	Asian American **(147)**	Asian American **(821) *****459%***
	Hispanic **(170)**	Hispanic **(272) *****60%***	Hispanic **(543)**	Hispanic **(671) *****24%***
	Whites **(137)**	Whites **(251) *****83%***	Whites **(268)**	Whites **(533) *****99%***
	Latino **(47)**	Latino **(210) *****347%***	Latino **(183)**	Latino **(530) *****190%***
	Person(s) of Color **(11)**	Person(s) of Color **(109) *****891%***	Person(s) of Color **(60)**	Person(s) of Color **(465) *****675%***
	Asian people **(43)**	Asian people **(185) *****330%***	Asian people **(80)**	Asian people **(498) *****523%***
	African American **(26)**	African American **(104) *****300%***	African American **(148)**	African American **(498) *****236%***
	Korean **(21)**	Korean **(77) *****267%***	Korean **(56)**	Korean **(311) *****455%***
	Japanese** (12)**	Japanese **(73) *****508%***	Japanese** (62)**	Japanese **(259) *****318%***
	Indian **(19)**	Indian **(76) *****300%***	Indian **(83)**	Indian **(241) *****190%***
	Black man (men) **(4)**	Black man (men) **(24) *****500%***	Black man (men) **(31)**	Black man (men) **(302) *****874%***
	Native American **(5)**	Native American **(18) *****260%***	Native American **(27)**	Native American **(153) *****467%***
	Mexican **(3)**	Mexican **(33) *****1000%***	Mexican **(54)**	Mexican **(130) *****141%***
	Black woman **(0)**	Black woman **(8) *****700%***	Black woman **(17)**	Black woman **(118) *****594%***
	Asian woman **(11)**	Asian woman **(33) *****200%***	Asian woman **(13)**	Asian woman **(106) *****715%***
	White man (men) **(3)**	White man (men) **(22) *****633%***	White man (men) **(30)**	White man (men) **(164) *****447%***
	White woman **(7)**	White woman **(13) *****86%***	White woman **(16)**	White woman **(105) *****556%***

## Footnotes

1 https://www.visualcapitalist.com/visualizing-u-s-population-by-race/.

2 https://www.census.gov/quickfacts/fact/table/US/PST045222.

3 https://covidtracking.com/race.

4 https://praw.readthedocs.io/en/latest/.

5 https://github.com/dmarx/psaw.

6 https://www.kdnuggets.com/2019/04/text-preprocessing-nlp-machine-learning.html.

7 https://spacy.io/api/annotation#biluo.

8 https://deepai.org/machine-learning-glossary-and-terms/precision-and-recall.

9 https://catalog.ldc.upenn.edu/docs/LDC2013T19/OntoNotes-Release-5.0.pdf.

10 manwar@ncat.edu.

11 https://www.healthypeople.gov/2020/topics-objectives/topic/social-determinants-of-health.
